# Single-Institution Experience in Clinical Trials During the COVID-19 Pandemic in Spain: Not So Bad After All?

**DOI:** 10.1200/GO.20.00247

**Published:** 2020-06-26

**Authors:** Paula Rodriguez-Otero, Joana Reis, Ana Alfonso-Pierola, Diego Salas-Benito, Miriam Giraldez, Jose Ramón Azanza, Mariano Ponz-Sarvise

**Affiliations:** Paula Rodriguez-Otero, MD, PhD, Department of Hematology, Clínica Universidad de Navarra, Pamplona, Spain; Joana Reis, Central Unit for Clinical Trials, Clínica Universidad de Navarra, Pamplona, Spain; Ana Alfonso-Pierola, MD, PhD, Department of Hematology, Clínica Universidad de Navarra, Pamplona, Spain; Diego Salas-Benito, MD, Department of Oncology, Clínica Universidad de Navarra, Pamplona, Spain; Miriam Giraldez, PharmD, PhD, Department of Pharmacy, Clínica Universidad de Navarra, Pamplona, Spain; Jose Ramón Azanza, MD, PhD, Department of Clinical Pharmacology, Clínica Universidad de Navarra, Pamplona, Spain; and Mariano Ponz-Sarvise, MD, PhD, Department of Oncology, Clínica Universidad de Navarra, Pamplona, Spain

## TO THE EDITOR:

The impact of the COVID-19 outbreak in Spain during March-April 2020 has been unbalanced throughout the different regions of the country. The alarm status defined by the government on March 14, and still in place at the time of this writing, has transformed the country in different perspectives, including care of patients with cancer.^1^ In many centers, clinical trial activity was suspended, because it was not considered a priority under the health care challenge of the COVID-19 pandemic.^2^ Nevertheless, experimental therapy is the only and/or best therapeutic option for many patients with cancer.

We performed a retrospective study to analyze the impact of the COVID-19 lockdown in the activity of our clinical trials unit. The analysis was restricted to hematology and oncology clinical trials. We analyzed two time periods: March 2, 2020, to April 19, 2020, and a comparable timeframe in 2019 (March 11, 2019, to April 28, 2019). Variables analyzed were number of patient visits, number of active patients, inclusion of new patients, and COVID-19 infection incidence.

At the beginning of the COVID-19 outbreak in Spain, we implemented a risk mitigation plan based on the Spanish Agency of Medicines and Medical Devices recommendations to reduce the impact of the pandemic in patients’ and health-workers’ security while maintaining treatment and quality of clinical trials. Three types of measures were established: patient oriented, sponsor oriented, and staff oriented.

Patient-oriented measures incorporated the following: (1) Individualized review of the scheduled on-site visits was made in advance to assess need: 41 (6%) of 641 visits were delayed; of these, 32 were treatment visits. In 10 instances, patients received oral medication at home after a remote clinical consultation. (2) COVID triage calls prior to the visit were performed by trained staff. In nine patients suspected or confirmed positive for SARS-CoV-2 (ie, severe acute respiratory syndrome, or COVID-19 infection), the on-site visit was cancelled. (3) A specific on-site patient route (“rapid intrahospital circuit”) was created: patients underwent a second triage, including temperature readings at the hospital entrance. In cases of suspected infection, SARS-CoV-2 polymerase chain reaction was performed in the COVID area. Surgical mask use was mandatory for every patient, as was polymerase chain reaction testing for those starting treatment. From a total of 298 patients (accounting for 600 on-site visits), only two were screened by PCR, and one had a positive result for COVID-19 infection (Table 1).

**TABLE 1:**
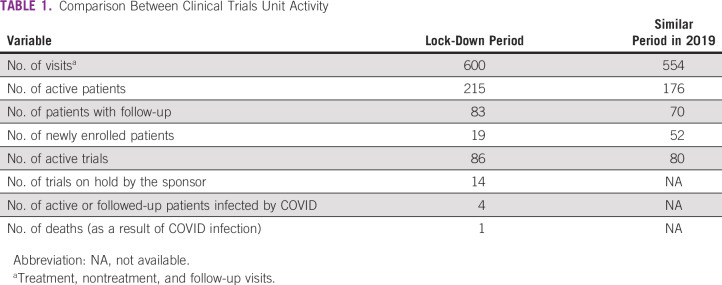
Comparison Between Clinical Trials Unit Activity

Sponsor-oriented measures were the following: (1) All on-site activities—including site-initiation, monitoring, closeout, and selection visits—were substituted by remote visits, thus guaranteeing the quality throughout the trials; (2) recruitment was paused for 2 weeks, because intensive care unit and hospitalization beds were overcrowded with patients being treated for COVID-19; and (3) active communications were held with sponsors every 2 weeks to provide updates on the situation.

Staff-oriented measures were as follows: (1) Shifts within the staff were organized and aimed at decreasing the risk of infection, and (2) among the 22 staff members, three physicians, four study coordinators, one data entry, one clinical assistant, and three laboratory technicians constituted the essential on-site staff. The remainder worked remotely.

The main results are summarized in Table 1. Overall, the clinical activity during the lockdown was maintained. The numbers of visits, active patients, and active trials were similar between the two periods analyzed. However, new patient enrollment was affected and significantly decreased (n = 19 *v* 52). Likewise, the number of trials held by sponsors during the COVID-19 outbreak was higher (n = 0 *v* 14). Only four of 215 patients developed SARS-CoV-2 infection, and none of them were infected at our hospital: one patient was treated at home, two were admitted at referral hospitals, and one was admitted at our site. Two patients died of COVID-19 infection.

The implementation of strict measures at three levels (patients, sponsors, and staff) allowed us to maintain a highly stable and secure activity in our clinical trials unit, which translated into an important benefit for patients with cancer.
